# Observation of Multi-Phonon Emission in Monolayer WS_2_ on Various Substrates

**DOI:** 10.3390/nano14010037

**Published:** 2023-12-22

**Authors:** Eli R. Adler, Thy Doan Mai Le, Ibrahim Boulares, Robert Boyd, Yangchen He, Daniel Rhodes, Edward Van Keuren, Paola Barbara, Sina Najmaei

**Affiliations:** 1 Department of Physics, Georgetown University, Washington, DC 20057, USA; era34@georgetown.edu (E.R.A.); tl780@georgetown.edu (T.D.M.L.); edward.van.keuren@georgetown.edu (E.V.K.); 2 U.S. Army Combat Capabilities Development Command, Army Research Laboratory, Adelphi, MD 20783, USA; 3 Department of Materials Science and Engineering, University of Wisconsin-Madison, Madison, WI 53706, USA

**Keywords:** two-dimensional materials, transition metal dichalcogenides, dark excitons

## Abstract

Transition metal dichalcogenides (TMDs) have unique absorption and emission properties that stem from their large excitonic binding energies, reduced-dielectric screening, and strong spin–orbit coupling. However, the role of substrates, phonons, and material defects in the excitonic scattering processes remains elusive. In tungsten-based TMDs, it is known that the excitons formed from electrons in the lower-energy conduction bands are dark in nature, whereas low-energy emissions in the photoluminescence spectrum have been linked to the brightening of these transitions, either via defect scattering or via phonon scattering with first-order phonon replicas. Through temperature and incident-power-dependent studies of WS_2_ grown by CVD or exfoliated from high-purity bulk crystal on different substrates, we demonstrate that the strong exciton–phonon coupling yields brightening of dark transitions up to sixth-order phonon replicas. We discuss the critical role of defects in the brightening pathways of dark excitons and their phonon replicas, and we elucidate that these emissions are intrinsic to the material and independent of substrate, encapsulation, growth method, and transfer approach.

## 1. Introduction

The intriguing electronic structure of monolayer transition metal dichalcogenides (TMDs) arises from the strong spin–orbit coupling and the lack of inversion symmetry, yielding a spin-splitting of the conduction and the valence bands at the K and K’ valleys of the hexagonal Brillouin zone, where the extrema of their direct bandgaps are located [1,2]. Such properties are unique to these materials and open exciting pathways towards novel spin/valley opto-electronics applications [3], especially in the realm of quantum computation [4,5]. However, such applications require a thorough understanding of the complex optical excitation and relaxation processes in these two-dimensional semiconductors. There are a variety of monolayer semiconducting TMDs (MX_2_, with M = Mo, W and X = S, Se and Te), with important differences in their optical transitions. Unlike molybdenum-based TMDs, the lower conduction band in tungsten-based TMDs carries an opposite spin to that of the upper valence band, making these lower energy, intra-valley transitions spin-forbidden. There are also momentum-forbidden transitions between conduction and valence bands with the same spin, but in different valleys. Nevertheless, in addition to the optically allowed bright transitions (e.g., neutral excitons and trions), the emission characteristics of monolayer tungsten-based TMDs show an increased level of complexity at lower energies, with a plethora of emissions that are not yet fully understood. These low-energy emissions have been broadly attributed to the brightening of initially dark excitons, either via defect or phonon scattering. Elastic scattering and binding by defects brighten these dark transitions by providing the spin-flip or the momentum change required by the selection rules, without the need of phonons [6,7,8,9]. This process gives rise to zero-phonon-line emissions, namely emissions with no phonon scattering [10,11,12,13]. Inelastic scattering with phonons, however, results in emissions that are replicas of the zero-phonon lines at energies shifted by an integer value of the phonon’s energy [10,11,12,13,14]. The strength of the electron–phonon coupling determines the specific phonons involved in the brightening of dark excitons [13]. Several emissions linked to inelastic scattering with phonons have been observed, including spin-flipping Γ phonons and spin-conserving K or Λ phonons [11,12,13,15]. In all cases, the emissions were attributed to first-order replicas, corresponding to single-phonon emissions.

It is clear that defects play an important role in the brightening of these dark excitons, but so far, the identities of the types of defects involved in the scattering process have been elusive. Recent studies included magneto-photoluminescence, resonant Raman, and photoluminescence spectroscopy on high-quality WS_2_ or WSe_2_ exfoliated from bulk crystals and, in some cases, encapsulated in hexagonal boron nitride (hBN) [10,11,12,13,15,16]. Even very clean samples, such as those encapsulated in hBN, have been argued to have defects that were intrinsic to the material, but could not be attributed to specific vacancies; therefore, questions on their origin have been left open [11]. The picture becomes more complicated for defects in larger-area materials grown by chemical vapor deposition (CVD) and for substrates other than hBN. The presence of substrate non-uniformity, as well as of the impurities left from the transfer process, may play a significant role in the brightening of dark excitons; however, systematic studies on the effect of the defect density in the material, of the encapsulation and of the interaction with the substrate are still lacking.

In this work, we present temperature, incident-power and polarization-dependent photoluminescence (PL) measurements on monolayer TMD flakes that are suspended or supported by a substrate. Specifically, we studied WS_2_ that was (a) exfoliated and encapsulated on hBN, (b) directly grown by chemical vapor deposition (CVD) on hBN and SiO_2_, and (c) grown by CVD and transferred onto SiO_2_ substrates patterned with holes. In both suspended and substrate-supported samples, a complex blend of low energy photoluminescence emissions is detected. The power and temperature dependence of these emissions, combined with Raman scattering information on the vibrational modes, suggest that the low energy emissions are defect-assisted phonon replicas, consistent with previously reported works [10,11,12,13,15]. More importantly, we observe multi-phonon emissions, with replicas up to the sixth order. The low-energy emissions are stronger and broader for samples transferred from the growth substrate, likely due to the larger number of defects/residues from the transfer process. These findings highlight important material design strategies that can be utilized to control excitonic dynamics in 2D van der Waals layers and advance the engineering tools needed to develop future 2D optoelectronic devices.

## 2. Materials and Methods

We examine five different types of WS_2_ samples: (1) CVD-grown monolayers on SiO_2_ (referred to as-grown), (2) CVD-grown monolayers on hBN, (3) CVT-grown bulk crystals exfoliated and encapsulated on both sides with hBN, (4) CVD-grown monolayers transferred to a fresh SiO_2_ substrate, and (5) CVD-grown monolayers transferred and suspended on holes that were pre-patterned in the SiO_2_ substrate. A visual summary of the samples is in the Supplementary Information in Figure S1. This comprehensive selection of WS_2_ materials and preparation methods allow us to examine samples with different defect densities (CVD-grown vs. CVT/exfoliated), as well as examine the role of substrates and their inhomogeneities, by comparing hBN, SiO_2_, and suspended samples.

The CVD growth is performed at atmospheric pressure (APCVD). Details on the precursors and substrate preparations are outlined in the supplementary information. For WS_2_ grown on top of hBN, the growth process is the same as the growth process for monolayer samples, with the main difference being the substrates for WS_2_ on hBN growth will have hBN exfoliated onto them via mechanical exfoliation, and then rinsed under electronic grade acetone followed by IPA to remove any tape residue.

The chemical vapor deposition process begins with an anneal for 60 min at 120 °C in an Argon environment with 500 sccm flow rate. The sulfur boat is heated to 85 °C throughout the anneal. Following the anneal, the temperature of the furnace is ramped up from 120 °C to 750 °C in 40 min and from 750 °C to 850 °C in 10 min. The Argon flow rate is maintained at 200 sccm from 120 °C to 750 °C and at 70 sccm from 750 °C to 850 °C, and throughout the growth phase. The temperature of the sulfur boat is increased from 85 °C to 175 °C throughout the temperature ramp phase in the heating profile. The Hydrogen flow rate is ramped from 0 to 10 sccm at 750 °C and maintained at this rate until the end of the growth phase. Once the WO_3_ boat reaches 750 °C, the sulfur temperature should be at 175 °C. At the end of the growth phase, the Hydrogen gas flow is turned off, the Argon flow rate is increased to 1000 sccm to flush out any residual by-products of the chemical reaction and the sulfur and furnace heating elements are turned off. The Argon gas flow is turned off once the furnace cools down below 200 °C.

For samples transferred from the growth substrate to a substrate patterned with holes, we used a water-assisted delamination process with polystyrene as a carrier layer [17]. Details on the transfer process are also included in the supplementary information.

Optical measurements from room temperature to 78 K were performed under high vacuum conditions, at 1.0 × 10^−6^ Torr, with a WITec Alpha 300RA system using the 532 nm line. The spectra were measured in the backscattering configuration using a 63× objective and an 1800 or 600 grooves mm^−1^ grating. The cooling was achieved with a SuperVariTemp Janis Research cryostat, using a continuous flow of liquid nitrogen as the cooling agent. This cryostat was adapted for Raman spectroscopy.

For temperatures below 78 K, we used a Montana Instrument cryostation equipped with a 100x objective in its chamber (Cryo-Optic, Bozeman, MT, USA) for high contrast imaging. The photoluminescence spectra were obtained by a single grating spectrometer (Horiba) and imaged on a CCD camera (Symphony I Horiba, Singapore). For laser sources, we employed a 532 nm diode and tunable M-squared laser (515 to 680 nm). 

## 3. Results

Optical excitation of WS_2_ comprises free charge carriers, neutral excitons (bright and dark), charged trions (bright and dark), as well as lattice vibrations (phonons). In the presence of free carriers, an exciton can undergo an electron exchange scattering event or bind a free carrier forming a trion [6,15,18,19,20,21,22]. Excitons and trions can interact with defects either by binding to a defect state or by elastic-scattering. The brightened dark excitons and their phonon replicas yield radiation emission and give rise to an extremely rich photoluminescence spectrum.

**Figure 1:**
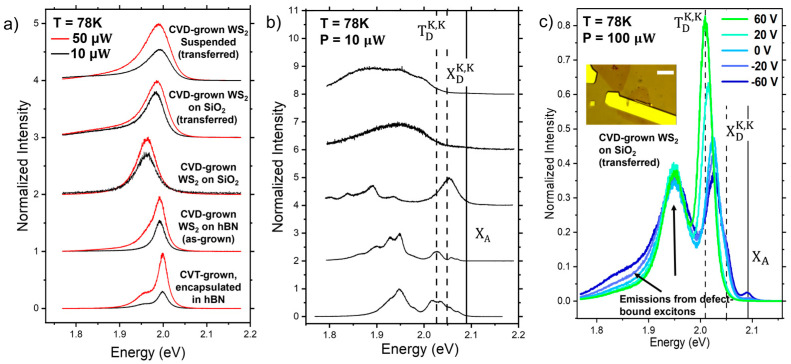
(**a**) Room temperature PL of different types of WS_2_ samples, namely (from top to bottom): CVD-grown WS_2_ monolayers on SiO_2_ transferred and suspended on top of pre-patterned holes on SiO_2_; CVD-grown WS_2_ monolayers transferred onto SiO_2_; CVD-grown WS_2_ monolayers on SiO_2_; CVD-grown WS_2_ monolayers on exfoliated hBN; CVT-grown bulk crystals exfoliated and encapsulated with hBN on both sides. (**b**) PL of the same set of samples as in (**a**) at 78K. (**c**) Gated-PL spectra of transferred WS_2_ on SiO_2_ at 78K. The vertical lines mark the peaks corresponding to transitions specified in Figure 2b. The inset shows an optical image of the gated sample. The scale bar is 20 µm.

Figure 1a shows the photoluminescence (PL) emission spectra from the five different samples at room temperature. The spectra are qualitatively similar for all of the samples, showing broad emissions in the same energy range. Notably, emissions can be better resolved in the spectrum collected from CVT WS_2_ on hBN, revealing more than one peak. This is expected, since cleaner materials and substrates with fewer defects and inhomogeneities lead to narrow emission linewidths, consistent with previous reports [6,8,11].

Figure 1b shows the PL spectra for the same samples at T = 78 K. These low-temperature spectra reveal a dramatic change relative to the room temperature emissions. The appearance of new peaks, the richer complexity of excitonic emissions, and the systematic variations between different samples point to interesting phonon- and defect-mediated processes. As discussed in previous work [13], at low temperature free excitons decay into lower-energy spin or momentum forbidden states, resulting in a relative decrease in the bright exciton peak and a relative increase in the low-energy dark exciton emissions. To help elucidate the specific origin of the PL emissions, we use electrostatic doping to modulate the charge carrier density. We used e-beam lithography and e-beam evaporation to pattern a gold contact on a CVD-grown WS_2_ monolayer that had been transferred to a doped silicon substrate covered with a SiO_2_ dielectric layer. The doped silicon was used as a back gate.

Since our CVD-grown WS_2_ is electron doped, a negative gate voltage depletes the excess electrons and enhances the neutral excitations. Figure 1c shows the photoluminescence spectra of WS_2_ on SiO_2_ at 78K at gate voltages ranging from +60 to −60 V, with 100 μW illumination and laser photon energy of 2.33 eV. We note that the curve corresponding to Vg = 0 in Figure 1c shows a prominent peak above 2.0 eV, in addition to the broad peak at about 1.9 eV that dominates the spectrum for the CVD sample transferred on SiO_2_ in Figure 1b. This is because the two spectra are measured with different incident power (the power is 10 times higher for the gated spectra in Figure 1c) and the relative peak height for different emissions strongly depends on the incident power, as it will be discussed later. The broader energy peak near 1.9 eV is prominent for samples transferred from the growth substrate and it has a very weak gate dependence. This peak is likely related to the presence of defects and residues from the transfer process, as outlined later in the discussion section. At negative gate voltages, the free exciton emission X_A_ emerges, with peak energy marked by the vertical line at about 2.1 eV.

**Figure 2:**
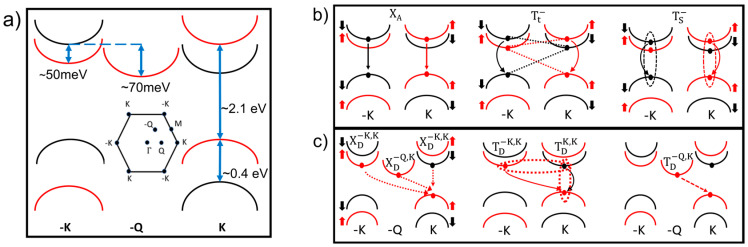
(**a**) Band alignment of WS_2_ for high symmetry points in the first Brillouin zone. The energy spacings correspond to T = 78 K. (**b**) Configurations of bright exciton and negatively charged trion (triplet and singlet) (**c**) Configurations of dark (spin and momentum forbidden transitions) excitons and trions.

Following the identification of the neutral exciton emission X_A_, the remainder of the peaks can then be identified by using the band diagram in Figure 2. The conduction band splitting gives rise to a spin-forbidden dark exciton X_D_^KK^ and momentum-forbidden dark exciton X_D_^-KK^. It has been reported that the momentum forbidden dark exciton X_D_^-KK^ lies about 10 meV higher than that of the spin forbidden, due to exchange effects [6]. In Figure 1c emissions corresponding to these transitions can be identified at approximately 50 meV below X_A,_ where the spectrum shows a shoulder. As expected for neutral excitons, this feature increases with the applied negative gate voltage, like X_A_ (see also Figure S2). We labeled this transition as X_D_^KK^, although, due to the close spacing between X_D_^KK^ and X_D_^-KK^, it is not possible to discern whether either transitions or both are contributing to this emission. The next prominent peak in Figure 1c) has a gate dependence characteristic of negatively charged trions. This emission is ~76meV below X_A_. This energy spacing from X_A_ is much larger than the ~20–35 meV additional binding energy of an extra electron expected for bright trions T_-_ [8,15,16,19,20,21,22], suggesting that the peak in Figure 1c is likely due to a negatively charged dark trion, indicated as T_D_^KK^ in Figure 2. In fact, T_D_^KK^ requires the additional binding energy corresponding to the splitting of the conduction band, energetically in agreement with the interpretation from magneto PL measurements in previous work [15].

**Figure 3:**
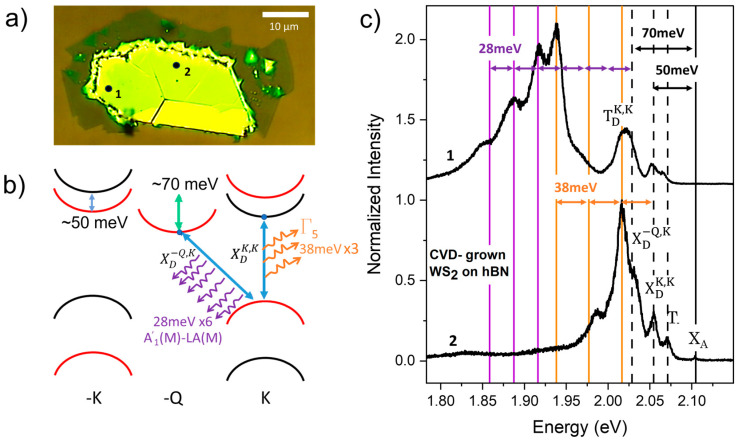
(**a**) Optical image of the WS_2_ flake grown on hBN (yellow) and SiO_2_ (brown). The dots labeled as 1 and 2 indicate the positions where the spectra in (**c**) were measured. (**b**) Band diagram showing phonon replicas corresponding to emissions in (**c**). The vertical transitions are spin forbidden and brightened by Γ_5_ phonons. The corresponding peak positions in (**c**) are marked by orange lines. The momentum indirect transitions correspond to three peaks marked by the purple lines with four, five and six A’1(m)-LA(M) phonons, respectively. (**c**) Spectra measured at two different points for the flake shown in (**a**), with 10 μW of incident power (532 nm) at 78K. (The top graph is one of the spectra in Figure 1b).

To better analyze the rich features of the lower energy spectra, Figure 3 shows a detailed analysis of the spectrum for the WS_2_ sample grown on hBN by CVD. The two PL spectra in Figure 3c are from the same flake, measured at two different locations. Although the low energy peaks can be found in all the samples, their intensity depends on the specific sample location. This is an example of the spectral emission variability within each flake that is also illustrated in the photoluminescence map in Figure S3. The spatial inhomogeneity confirms that these peaks are related to the presence of defects in the materials and may be stronger in regions with higher defect densities.

In Figure 3c, by marking the emission from the neutral excitons X_A_, we can clearly measure the energy spacing of the other emissions relative to X_A_. We note that the peaks corresponding to X_A_ (2.1 eV) and the bright trions T_-_ (2.068 eV) are the weakest peaks, while the low-energy emissions dominate the spectrum. Several recent studies have attributed these low-energy emissions to the brightening of dark excitons [6,7,8,11,12,15,16,23,24], but clearly identifying the corresponding transitions and their brightening mechanism are challenging tasks, often leaving behind open questions. For the different types of samples described here, we already described above how we identify the zero-phonon lines of dark transitions X_D_^KK^ and T_D_^KK^. As mentioned in the discussion of the spectra in Figure 1, X_D_^KK^ is spaced from X_A_ by 50 meV, approximately the conduction band splitting, consistent with the zero-phonon line of the spin-forbidden dark exciton brightened by elastic scattering with defects. This vertical transition can also be brightened by a Γ phonon via spin-flip scattering. For WS_2_ this is the Γ^5^ phonon, corresponding to the in-plane out-of-phase oscillation of the chalcogen atoms with an energy of about 38 meV [25]. The emissions marked by the vertical orange lines correspond to peak energies matching the first- and the third-order phonon replicas for these phonon-brightened transitions in the lower and upper spectrum, respectively.

Similarly, we can identify the zero-phonon line of the momentum-forbidden transitions X_D_^-QK^ (marked by a dotted line just above 2.025 eV) between electrons and holes in the -Q and K bands, respectively. The energy spacing of this peak from X_A_ matches the spacing of the conduction band edges at -Q and K, about 70 meV, indicating that the brightening of the X_D_^-QK^ zero-phonon line is due to elastic scattering with defects. These transitions can also be brightened by scattering with phonons carrying the matching momentum difference between the electron and the hole. These are acoustic phonons at the M point of the Brillouin zone that have been shown to strongly couple to excitonic transitions in previous experiments using resonant Raman spectroscopy [13]. In our spectra we can clearly see peaks with spacing of 28 meV matching the A1′(M)-LA(M) phonon for WS_2_. Notably, the spacing of the peaks from the zero-phonon line indicates that we can measure up to the sixth-order phonon replica for this phonon-brightened emission, as indicated by the purple dotted lines. Momentum conservation requires either just one phonon or an odd number of phonons that includes pairs of phonons with opposite momentum. The presence of replicas corresponding to an even number of phonons indicates that elastic scattering with defects must contribute to these emissions to conserve momentum. This is consistent with the spatial non-uniformity of the intensity of these low-energy emissions. It is also interesting to note that there are regions of the spectra where several different emissions are closely spaced. For example, in the spectrum corresponding to location 2 in Figure 3c, the peaks from the first Γ^5^ replica of X_D_^KK^, from the zero-phonon line X_D_^-QK^ and from the dark trion T_D_^KK^ are all located in the region of the spectrum where the emission is strongest, at 2.02 eV.

**Figure 4:**
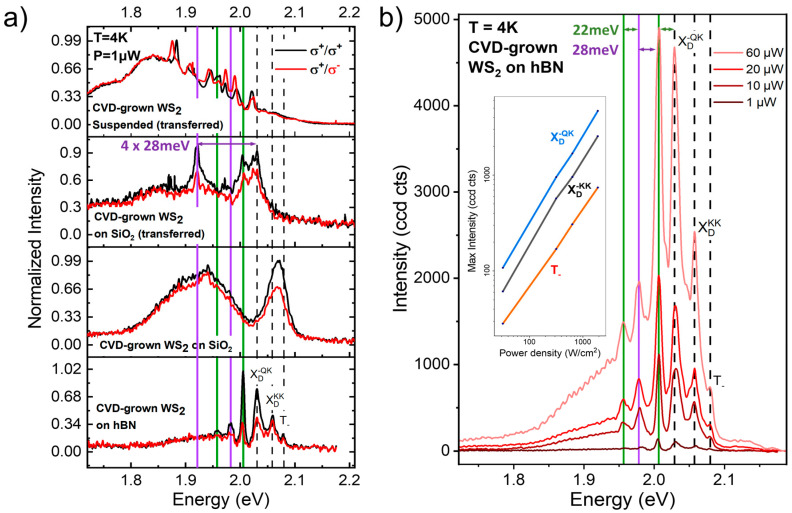
(**a**) Helicity-resolved PL spectra taken at 4K from various WS_2_ monolayer samples. (**b**) PL emissions at various powers under unpolarized excitation laser.

Polarization dependence of the PL spectrum is a strong tool to study the character of emissions and their origins. Figure 4a shows the co- and cross-polarized spectra from the different WS_2_ samples at 4K. For the suspended samples and the samples on the SiO_2_ substrates, the emission is characterized by a broad peak as a background to sharper emissions. The broad peak is less prominent for the sample on hBN. While the broad peak is polarization independent, the sharper emission peaks do change with polarization, as expected for bright excitons and dark excitons that are not bound to defects. The lack of polarization dependence of the broad background peak suggests that its origin might be from excitons bound to defects, rather than excitons scattered by defects [26,27,28,29,30]. The sharper peaks can be more clearly distinguished in spectra with higher power in Figure 4b, where vertical lines are used to identify the zero phonon lines and the corresponding replicas for the more prominent peaks, although small shoulders between the peaks reveal the presence of other emissions with smaller intensity, such as the zero-phonon line T_D_^KK^. The peaks identified at energy lower than the zero-phonon line X_D_*^-^*^QK^ are replicas with spacings of about 22 meV, corresponding to the LA(M) phonon mode [13] and 28 meV, corresponding to the A1′(M)-LA(M) phonon energy [25].

Figure 4b clearly shows that the relative peak height from different emissions changes as a function of power. Specifically, the peak heights of the zero-phonon lines and the phonon replicas continue to grow with power approximately linearly, while the broad defect background saturates. These results indicate that the broad background is due to defect-bound excitons, with emissions that are expected to saturate with power for a finite defect density, once all the defect traps are occupied by excitons. On the other hand, the zero-phonon lines and the phonon replicas for dark emissions are brightened by elastic scattering of excitons with defects; therefore, the peak height continues to increase with increasing incident power.

## 4. Discussion

The low energy features in the photoluminescence spectra of all the different samples studied here show clear common features. They develop at low temperatures, and they dominate the spectrum at low incident laser power. This is evident from the maps of the PL spectra. At room temperature, where the emission is typically dominated by the bright emissions, i.e., by the trion peak (for CVD-grown material, typically electron-doped due to sulfur vacancies), the emission is spatially very uniform. At low temperature, the spatial non-uniformity emerges, and the low-energy emissions are stronger in some regions of the flakes, as shown, for example, by the two spectra in Figure 3c (see also the photoluminescence map in the supplementary information). We use the analysis of the spectra from different types of samples combined with the studies of the spectra as a function of electrostatic gating to identify the low energy emissions of dark excitations either as zero phonon lines or as their related phonon replicas. The spatial non-uniformity of the spectrum points to the essential role played by defects in the brightening process. Since the zero-phonon lines match the energy spacing from the neutral exciton emission X_A_ that is expected from the corresponding dark transition in the WS_2_ band diagram (see Figure 2), the defect-assisted brightening must be the result of elastic scattering, rather than emissions from excitons bound to defects observed in previous works, where the energies of the low-energy emission zero phonon lines were determined by the defect binding energies [11]. In addition, we can clearly identify peaks from phonon replicas of the zero-phonon lines by matching their spacing to WS_2_ phonons that satisfy the energy and momentum requirements to brighten these dark transitions. For the brightening of momentum-indirect dark transitions, we identify zone-edge M phonons that yield emissions up to sixth-order replicas of the corresponding zero-phonon line. This result is consistent with previous observations of strong exciton–phonon scattering intensity for M phonons in WS_2_ [13]. Our work elucidates that this exciton–phonon coupling is not strongly affected by the substrate, since these low-energy multi-phonon replicas can be identified in samples with SiO_2_ and hBN substrates, as well as in suspended samples, as shown in Figure 4a. It is interesting to note that in semiconductors the intensity of phonon replicas with multi-phonon emission can be related to the electron–phonon coupling via the Huang-Rhys factor [14,31,32]. Although this work is mainly focused on identifying the phonon replicas and their occurrence for different types of samples, in future work we plan to study the intensities of the phonon replicas for different transitions and different phonon modes to extract Huang-Rhys factors for WS_2_ and other transition metal dichalcogenides.

By comparing the spectra from different types of samples, we find that the effects of the substrate and the defect density in the WS_2_ are manifest mainly in the broadening of these emissions. As expected, more uniform substrates and low- defect density samples yield sharper emission lines. The broader low-energy emissions are measured from the transferred samples (see for example transferred CVD WS_2_ in Figure 1b), indicating higher defect density from residues related to the transfer process. However, measurements at lower temperature in Figure 4, show that sharp phonon replicas emerge from the broad emission background, confirming that phonon replicas are present also in the transferred samples. While the phonon replicas and the zero-phonon lines are polarization-dependent and indicate brightening of dark excitons that is assisted by elastic scattering with defects, the broad polarization-independent background is indicative of excitons bound to defects. Unlike emissions brightened by elastic scattering, emissions from excitons bound to defects saturate with increasing power, as shown in Figure 4b.

## 5. Conclusions

In summary, we show that the low-energy emissions in monolayer WS_2_ are largely governed by scattering with phonons and elastic scattering from defects, which remain regardless of the substrate or growth conditions. In addition, the power dependence and the spatial non-uniformity confirm that defects seem to play a key role in the occurrence of radiative recombination from dark excitons. However, these defects are not related to the substrate or different defect densities from different growth processes, since these low energy emissions are ubiquitous for samples on different substrates, grown and prepared with different methods. In our analysis, evidence of emissions due to defect-bound exciton described in previous work [11] was limited to a broad background signal that was prominent in samples with residues from the transfer process. Our findings highlight important physics and material design strategies that can be utilized to control excitonic dynamics in 2D van der Waals layers and advance the engineering tools needed for the development of 2D optoelectronic devices.

## Data Availability

Supporting data will be shared upon request.

## Supplementary Materials

The supporting information can be downloaded at: https://www.mdpi.com/article/10.3390/nano14010037/s1.
