# Supplementary material for: Observation of Multi-Phonon Emission in Monolayer WS2 on Various Substrates
Source: Nanomaterials (Basel). 2023 Dec 22;14(1):37. doi: 10.3390/nano14010037 (PMC10780943; doi:10.3390/nano14010037)
Supplement: Supplementary file 1 [file nanomaterials-14-00037-s001.zip › nanomaterials-2769493-supplementary.pdf]

nanomaterials

Type of the Paper: Article

Observation of Multi-Phonon Emission in Monolayer WS_2 on Various Substrates

Eli R. Adler [1,2], Thy Doan Mai Le [1], Ibrahim Boulares [2], Robert Boyd [3], Yangchen He [3], Daniel Rhodes [3], Edward Van Keuren [1], Paola Barbara [1,*] and Sina Najmaei [2,*]

1 Department of Physics, Georgetown University, Washington, DC 20057, USA; era34@georgetown.edu (E.R.A.); tl780@georgetown.edu (T.D.M.L.); edward.van.keuren@georgetown.edu (E.V.K.)
2 U.S. Army Combat Capabilities Development Command, Army Research Laboratory, Adelphi, MD 20783, USA
3 Department of Materials Science and Engineering, University of Wisconsin-Madison, Madison, WI 53706, USA
* Correspondence: paola.barbara@georgetown.edu (P.B.); sina.najmaei.civ@army.mil (S.N.)

Supplementary information

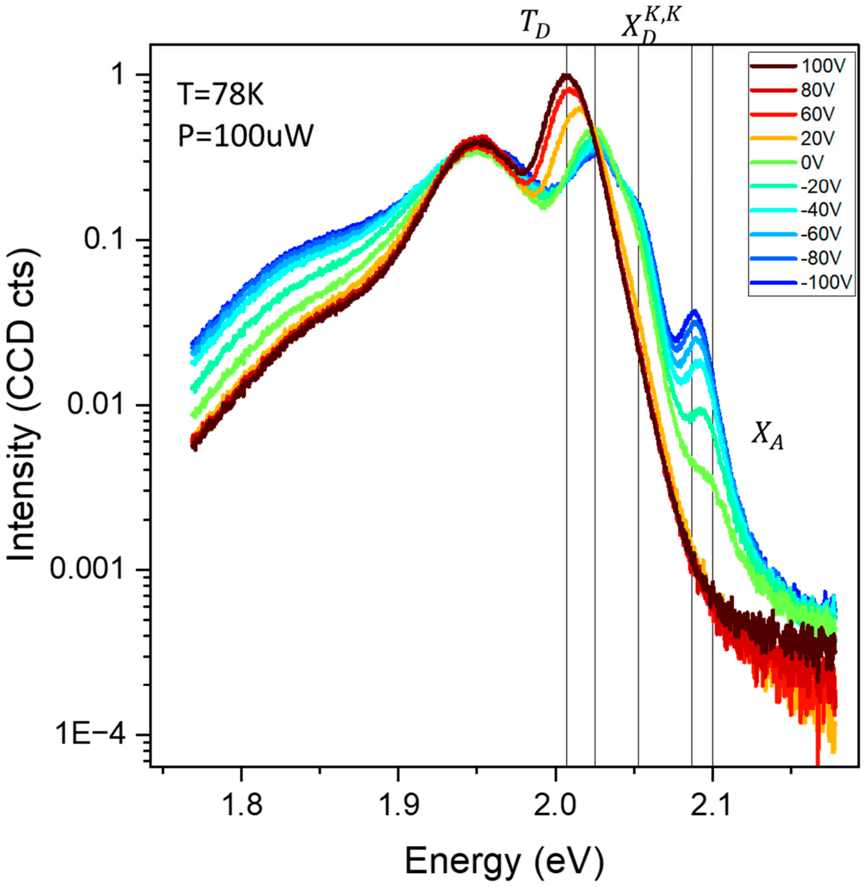

Figure S1 Semi-log scale of gated-PL measurement at 78K (figure 1c in main text)

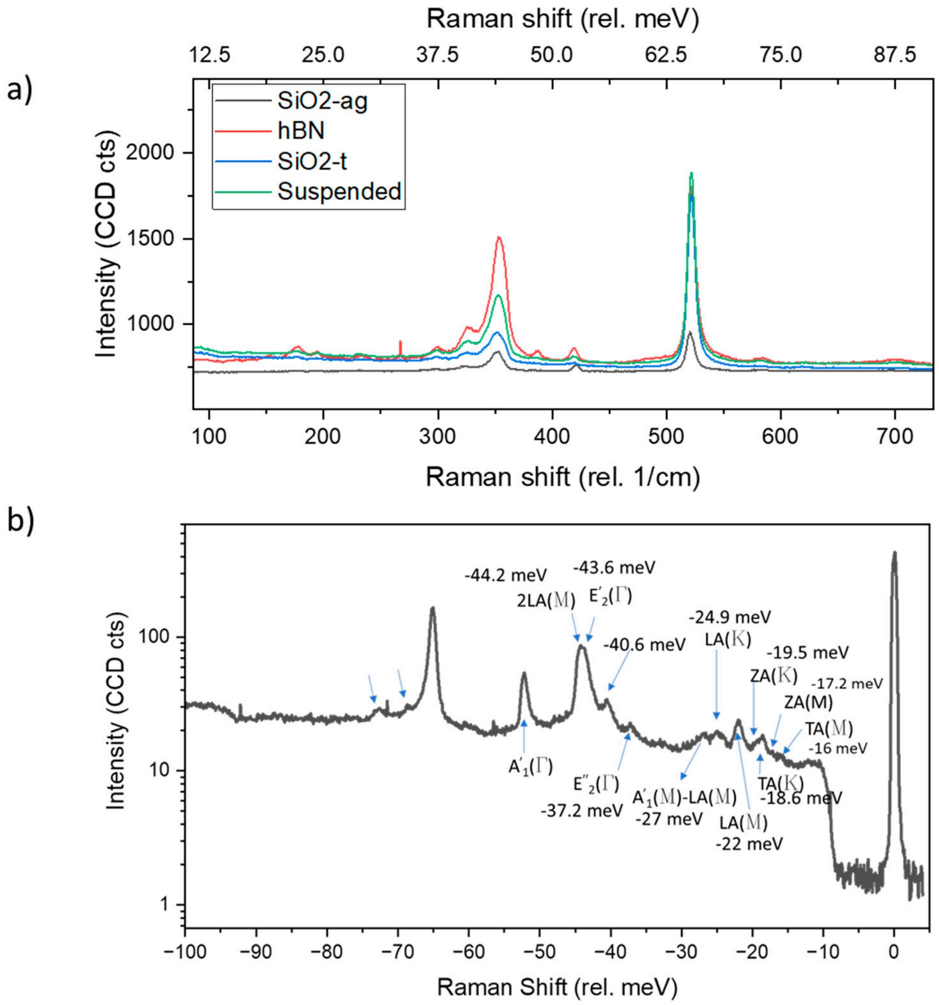

Figure S2 a) Room-temperature Raman measurements of WS_2 on various substrates. b) Raman measurement of WS_2 grown on hBN

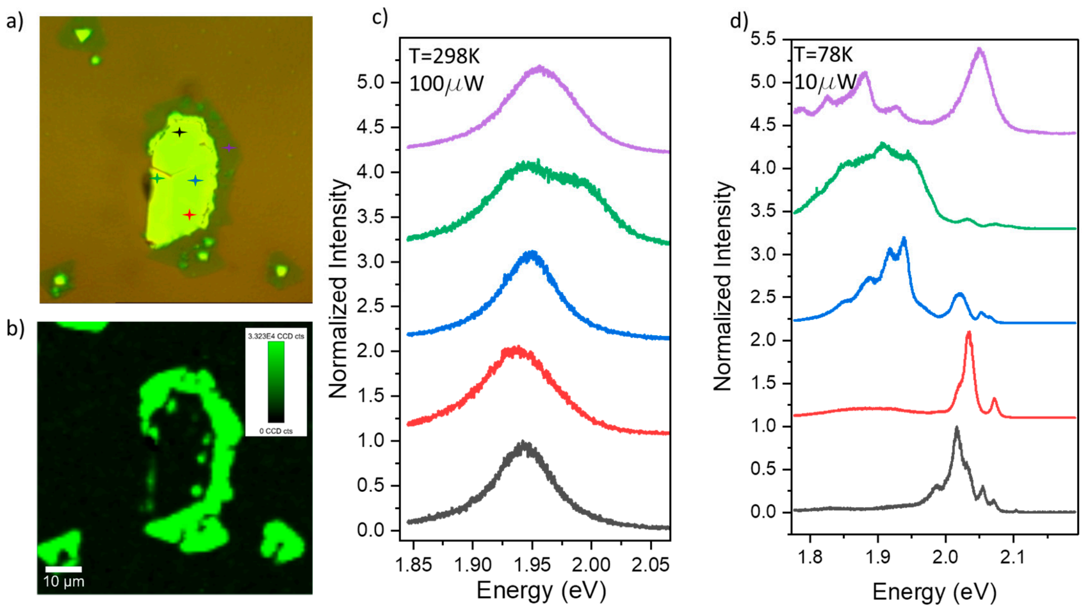

Figure S3 Spatial variations of PL spectra. a) optical image of WS_2 grown on hBN with colored markers corresponding to c) and d). b) Integrated PL spectra vs position. c) Spatial variations in PL with colors indicated by markers in a)

CVD Growth of 1L WS_2

The chemical vapor deposition of monolayer WS_2 begins with preparing the solid precursor powders and treating the target substrates with piranha solution. For monolayer WS_2, our recipe calls for 0.0001g of 99.99% WO_3 powder (Millipore Sigma) to be spread evenly at the bottom of the alumina boat, and 0.3g of S powder to be packed into the quartz boat. The target SiO_2/Si substrates are first cleaved into 7 pieces from a larger wafer, each piece measuring roughly 0.5cm by 2cm, and then cleaned in a piranha solution (3 volumetric parts of H_2SO_4 by 1 volumetric part of H_2O_2 for 30 minutes, followed by 3 deionized water baths of 5 minutes each. The target substrates would then dehydrate on top of a 65°C hot plate for 30 minutes. Once dry, 15**μ**m of 4% perylene-3,4,9,10-tetracarboxylic acid tetrapotassium salt (PTAS) promoter is drop-casted on top of 3 target substrates, each, while on the hotplate using a pipette. The substrates are left to air dry on the hotplate until all promoter solution has air dried. Then, the substrates are suspended across the rim of the alumina boat, with the polished side facing the precursor powder at the bottom of the alumina boat. Once prepped, the alumina boat is loaded into the quartz tube, followed by the sulfur boat. The alumina boat remains in a zone heated by the furnace, whereas the sulfur boat is stationed away from the furnace heating elements and is heated separately, due to sulfur's much lower sublimation point compared to that of WO_3.

For WS_2 grown on top of hBN, the growth process is the same as the growth process for monolayer samples, with the main difference being the substrates for WS_2 on hBN growth will have hBN exfoliated onto them via mechanical exfoliation, and then rinsed under electronic grade acetone followed by IPA to remove any tape residue.

The chemical vapor deposition process begins with an anneal for 60 minutes at 120°C in an Argon environment with 500sccm flow rate. The sulfur boat is heated to 85°C throughout the anneal. Following the anneal, the temperature of the furnace is ramped up from

120°C to 750°C in 40 minutes and from 750°C to 850°C in 10 minutes. The Argon flow rate is maintained at 200sccm from 120°C to 750° C and at 70sccm from 750°C to 850°C, and throughout the growth phase. The temperature of the sulfur boat is increased from 85°C to 175°C throughout the temperature ramp phase in the heating profile. The Hydrogen flow rate is ramped from 0 to 10sccm at 750°C and maintained at this rate until the end of the growth phase. Once the WO_3 boat reaches 750°C, the sulfur temperature should be at 175°C. At the end of the growth phase, the Hydrogen gas flow is turned off, the Argon flow rate is increased to 1000sccm to flush out any residual by-products of the chemical reaction and the sulfur and furnace heating elements are turned off. The Argon gas flow is turned off once the furnace cools down below 200°C.

Transfer of CVD grown material

We fabricate suspended WS_2 samples using a polystyrene-assisted wet etch transfer method to transfer the TMD onto the target substrates[1]. A layer of 10:1 toluene-to-polystyrene (260000g/mol polystyrene) solution is spun coat on top of the as-grown substrates at 3000 RPMs for 60s. The substrates are then baked at 85°C for 1 hour and then scratches at the edges of the substrates for the wet etchant to better access the SiO_2 layer. The substrates are then deposited on the surface of a Buffered Oxide Etch (Transene) bath for a minimum of 24 hours. BOE is a glass etchant, thus it will etch away the SiO_2 layer of the floating stack and release the polymer/TMD portion of the stack as a floating film at the end of the etch. At that point, the polymer/TMD film can be removed from the BHF bath and transferred to 3 consecutive deionized water baths, each 20 minutes long, to remove as much residual BOE as possible. BHF can p-dope the 2D material quite easily thanks to the abundant H+ ions in solution. Once the water baths are complete, a piranha-solution-treated dry thermal oxide SiO_2/Si substrate is used to scoop the polymer/TMD films out of the water bath and the substrate/film complex is then dehydrated via an 80°C bake on a hot plate for 60 minutes. Following the soft bake, the transferred substrates are then hard baked at 150°C for 30 minutes to partially melt the polymer and release the TMD from the polymer. After the hard bake, the transferred substrates are submerged in an acetone bath at 70°C to remove the polystyrene and toluene, followed by an IPA rinse.

References

[1] A. Gurarslan *et al.*, "Surface-energy-assisted perfect transfer of centimeter-scale monolayer and few-layer MoS2 films onto arbitrary substrates," *ACS nano,* vol. 8, no. 11, pp. 11522-11528, 2014.
